# 3D‐Printed Gastrointestinal Stents: In Vivo Evaluation in a Swine Small Bowel Perforation Model

**DOI:** 10.1002/adhm.202505169

**Published:** 2025-12-26

**Authors:** Gweniviere Capron, Parinaz Fathi, Tor Wolf Jensen, Derek J. Milner, André J. van der Vlies, Regan L. Moody, Elizabeth A. Bangert, Blair Rowitz, Dipanjan Pan, Matthew B. Wheeler

**Affiliations:** ^1^ Carle Foundation Hospital Urbana IL USA; ^2^ Department of Bioengineering University of Illinois at Urbana‐Champaign Urbana IL USA; ^3^ Materials Science and Engineering University of Illinois at Urbana‐Champaign Urbana IL USA; ^4^ Beckman Institute University of Illinois at Urbana‐Champaign Urbana IL USA; ^5^ Cancer Center at Illinois University of Illinois at Urbana‐Champaign Urbana IL USA; ^6^ Institute For Genomic Biology University of Illinois at Urbana‐Champaign Urbana IL USA; ^7^ Department of Animal Sciences University of Illinois at Urbana‐Champaign Urbana IL USA; ^8^ Carle Illinois College of Medicine University of Illinois at Urbana‐Champaign Urbana IL USA; ^9^ Materials Science and Engineering Pennsylvania State University University Park PA USA; ^10^ Departments of Biomedical Engineering Nuclear Engineering, and Materials Science and Engineering Pennsylvania State University University Park PA USA; ^11^ Huck Institutes of the Life Sciences, 101 Huck Life Sciences Building University Park PA USA

**Keywords:** stent, small bowel perforation, swine model

## Abstract

Gastrointestinal fistulae and perforations can lead to severe complications including sepsis and patient death. Implantation of personalized gastrointestinal stents can prevent leakage and ameliorate complications, without requiring removal post‐healing. In this work, the efficacy of 3D‐printed gastrointestinal stents composed of poly‐lactic‐acid (PLA) is evaluated in an in vivo swine model. Custom stent dimensions are determined for each subject using computed tomography imaging, and stents are implanted after an intestinal incision is made. A 1‐cm intestinal defect is maintained over the stent surface to evaluate the ability of the stents to retain intestinal contents over a span of two weeks. Stent efficacy is evaluated after necropsy by histology and scanning electron microscopic analysis. Stents were found to prevent abdominal sepsis over the two‐week period, even in the presence of an intestinal defect.

## Graphical abstract

Gastrointestinal fistulae and perforations can lead to severe complications including sepsis and patient death. In this work, the efficacy of 3D‐printed gastrointestinal stents composed of poly‐lactic‐acid (PLA) was evaluated in an in vivo swine model. Custom stent dimensions were determined for each subject using computed tomography imaging, and stents were implanted. Stent efficacy was evaluated after necropsy by histology and scanning electron microscopic analysis.

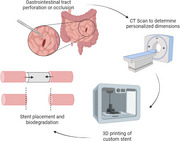

## 1 Introduction

Enterocutaneous fistula is an abnormal connection that can form between the intestinal tract or stomach and the skin [1, 2]. These are feared complications of surgical procedures such as small bowel anastomoses, which involves the joining of two remaining sections of the colon after bowel resection [3]. There are many situations that may require a small bowel resection, the removal of part of the intestine, including small bowel obstruction [4], inflammatory bowel disease [5], ischemia [6], iatrogenic injury [7] or perforation [8]. These situations are often fraught with peril as there is always a risk of breakdown of the anastomosis which can lead to intra‐abdominal sepsis, a life‐threatening reaction of the body to an infection [9] or enterocutaneous fistula. This risk of anastomotic break down and enterocutaneous fistula formation increases as the amount of inflammation in the area increases. The alternative to creating a primary anastomosis is to perform an enterostomy to make a stoma, a connection between the bowel and the skin on the outside of the body, however these come with their own problems [10, 11, 12]. In obese patients, they can be difficult to construct, challenging to manage postoperatively, and carry a risk of dehydration [12] and skin excoriation around the stoma area [13]. Furthermore, enterocutaneous fistula and stoma will all require at least one additional surgery to reverse [14, 15].

Enterocutaneous fistula are a serious problem with many possible complications. The morbidity of these fistula includes skin excoriation [2], dehydration [16], and sepsis [1], while the mortality from sepsis is generally 5%–20% [1, 17, 18, 19, 20]. Approximately 25% of enterocutaneous fistula will heal with non‐operative management, however the rest require a significant surgical intervention to correct them [21]. Current management consists of delaying surgical repair in order to manage inflammatory, infectious, nutritional, and wound healing complications. This process commonly involves extended patient hospitalizations/frequent readmissions, prolonged parenteral nutrition dependency, profound electrolyte deficiencies, and extensive wound management requirements. Definitive surgical repair is generally delayed for at least 6–12 months to allow for intraabdominal inflammation and adhesions to resolve, resulting in very significant patient morbidity and mortality as well as sizable costs to the healthcare system [21]. Of note, there have been no significant innovations to improve surgical intervention options for small intestinal perforations in over 40 years.

Currently, there are no tools to help protect a small bowel anastomosis during healing without leaking [22] or developing into an enterocutaneous fistula. There are multiple tools that have been used in colon or esophageal perforations to assist with healing including stents [23, 24]. These stents are generally made of metal wire, covered with a polymer layer, and therefore not biodegradable. Degradable stents made of the biodegradable polymers poly(lactic acid) and poly(dioxanone) for treating benign esophageal stenosis [25] and refractory benign esophageal strictures [26] respectively have been reported. These stents are deployed endoscopically and then retrieved after the esophagus [23, 27] or colon [28, 29] has healed. These available stents allow diversion of luminal contents through the stent to bypass the leaks and effectively reduce inflammation, infectious complications, and fluid losses while simultaneously allowing for continued oral nutrition, in most cases allowing the gastrointestinal tract to heal without further surgical intervention. Although these stents work well in the esophagus and colon, they are not a viable treatment option for small bowel perforations because the small bowel is too distant from either the mouth or anus for endoscopic deployment.

**FIGURE 1:**
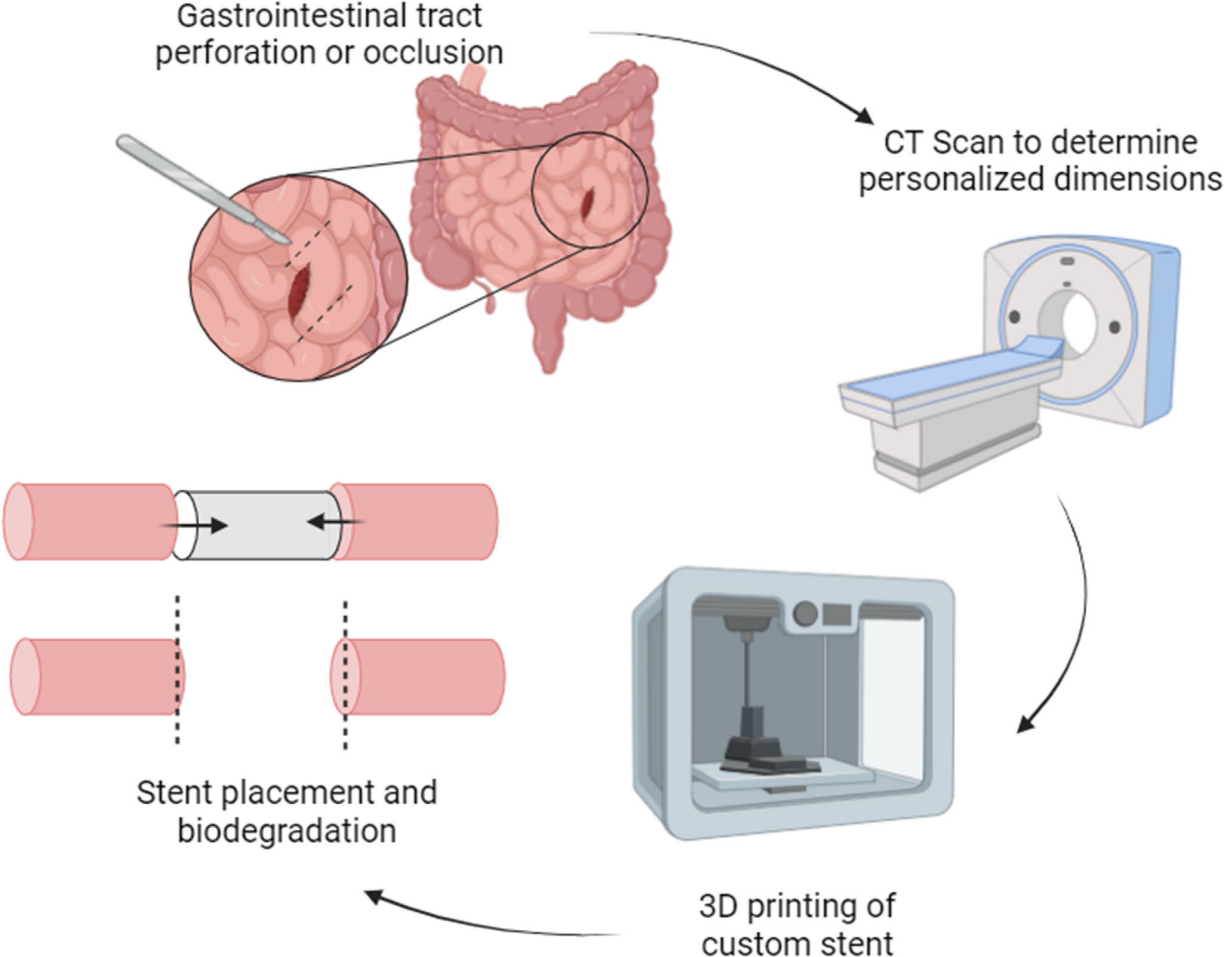
Development and implantation of 3D‐printed personalized biodegradable stent.

A biodegradable stent that could be placed at time of operation would not need a second surgery procedure. The ideal stent would protect the small bowel anastomosis from the intraluminal contents and allow the small bowel to heal, then degrade over time without need for surgical retrieval (Figure 1). Previously, we reported the development of 3D‐printed gastrointestinal stents composed of poly(caprolactone) (PCL) and poly(dioaxanone) (PDS) [21], both of which are FDA‐approved biodegradable polymers. The use of 3D‐printed stents allows for the rapid fabrication of customized stents using dimensions obtained from computed tomography (CT) images. Recently, the use of 3D printing for development of fistula stents has been demonstrated [30, 31].

In this work, we present in vivo efficacy and short‐term safety results for placement of 3D‐printed gastrointestinal stents in swine models of small intestine perforation [32]. A range of physiologically relevant stent sizes were determined from CT imaging, and stents were 3D‐printed with poly(lactic acid) (PLA). PLA is an FDA‐approved biodegradable polymer and is widely used for biomedical applications including drug delivery [33], tissue engineering [34, 35] and surgical sutures [36]. Degradation of PLA takes place via hydrolytic processes, resulting in lactic acid and oligomers that can be readily excreted from the body by renal clearance [36, 37, 38]. PLA has a median half‐life of 30 weeks [39]. PLA is commercially available in a variety of filament diameters for use in standard 3D printers [40]. We evaluated the ability of these stents to prevent leakage from an induced intestinal perforation in the small bowel.

## 2 Results

All four pigs survived until the planned euthanasia at 2 weeks without significant intervention or antibiotics. All pigs generally recovered well, were active and were tolerating food post operatively. Pig 1 had a normal post‐operative course aside from dark stools on post‐operative days 10–12. Pig 2 had watery stool noted on post‐operative days 3 and 5, with dark stools on post‐operative days 10 and 11. Pig 3 was noted to have dark stools on post‐operative days 10 and 11 as well. Pig 4 developed a fever to 39.4°C on post‐operative day 1, which had improved to 38.6°C on post‐operative day 2 and resolved by post‐operative day 3. It also had dark stools on post‐operative days 10–11, followed by lethargy and watery stools on post‐operative day 13.

**FIGURE 2:**
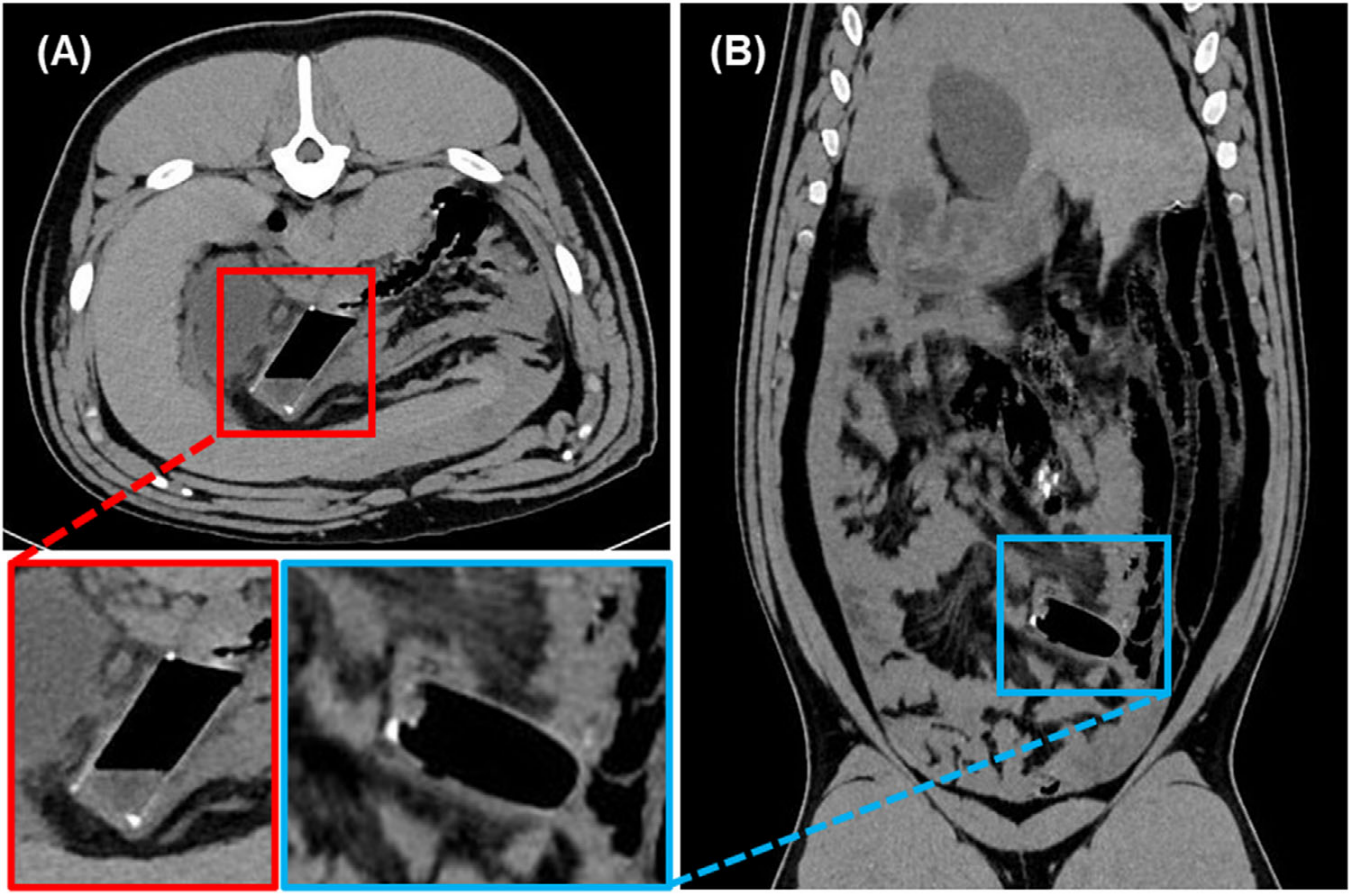
Representative CT images showing stents one‐week post‐placement. (A) Transverse view. (B) Coronal view. Observation of vascular clips confirms that the stent did not migrate from their initial positions.

CT scans performed at one week post stent placement demonstrated all stents in appropriate place (Figure 2). Vascular clips had been used to tag both the stents as well as suture outside of the bowel, and scans showed these clips closely approximated, which indicated the stents remained in place.

**FIGURE 3:**
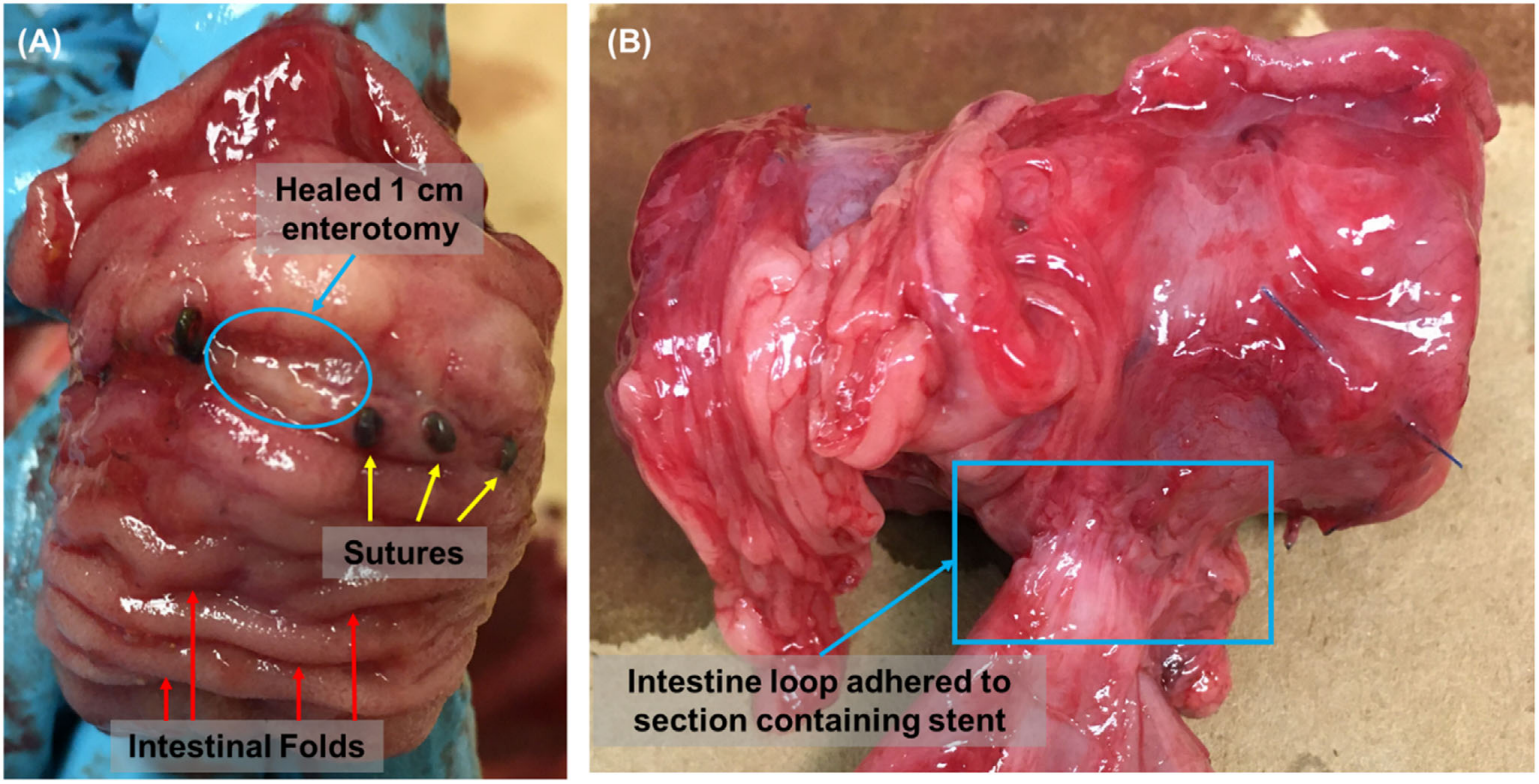
Representative images upon necropsy. (A) Evaluation of the 1 cm enterotomy site through turning the inside of the intestine outward. The 1 cm enterotomy has healed without fistula. Sutures and intestinal folds can be observed on the inner surface of the intestines. (B) Adhesion of an intestinal loop to the intestinal segment containing the stent.

Upon necropsy, the site of the 1 cm enterotomy appeared to have completely healed (Figure 3A). The abdomens were found to have moderate adhesions throughout with significant adhesions surrounding the enterotomy at the site of the stent placement. No signs of abscess or bowel leaks were found in any of the pigs. In three out of the four pigs, a loop of small bowel was adherent to the enterotomy site (Figure 3B); in the last pig the enterotomy site was adherent to the stomach. There was no sign of fistula formation. In the first three pigs, the stents had remained secured in place. In the last pig, the stent had migrated 5 cm down the small bowel lumen away from the enterotomy although it was unclear if manipulation during the necropsy had caused the stent to move or if the stent had become misplaced prior to the necropsy. A summary of surgical results is provided in Table 1.

**TABLE 1:** Summary of surgical results.

Pig	Measured Bowel Size^a^ (Calculated Diameter) [41]	Maximum Stent Outer Diameter [41]	Post‐ Operative Course	Abscess	Leakage	Fistula	Stent Migration
**1** **ID #2330**	26 (16.6)	21.8		No	No	No	No
**2** **ID #2101**	25 (15.9)	21.8	Watery stool POD 3 and 5	No	No	No	No
**3** **ID #2352**	25 (15.9)	23.22		No	No	No	No
**4** **ID #2372**	30 (19.1)	25.4	Fever on POD 1	No	No	No	Yes

^a^ Bowel size was measured with bowel flattened (i.e. dimension measured was approximately half of the circumference).

**FIGURE 4:**
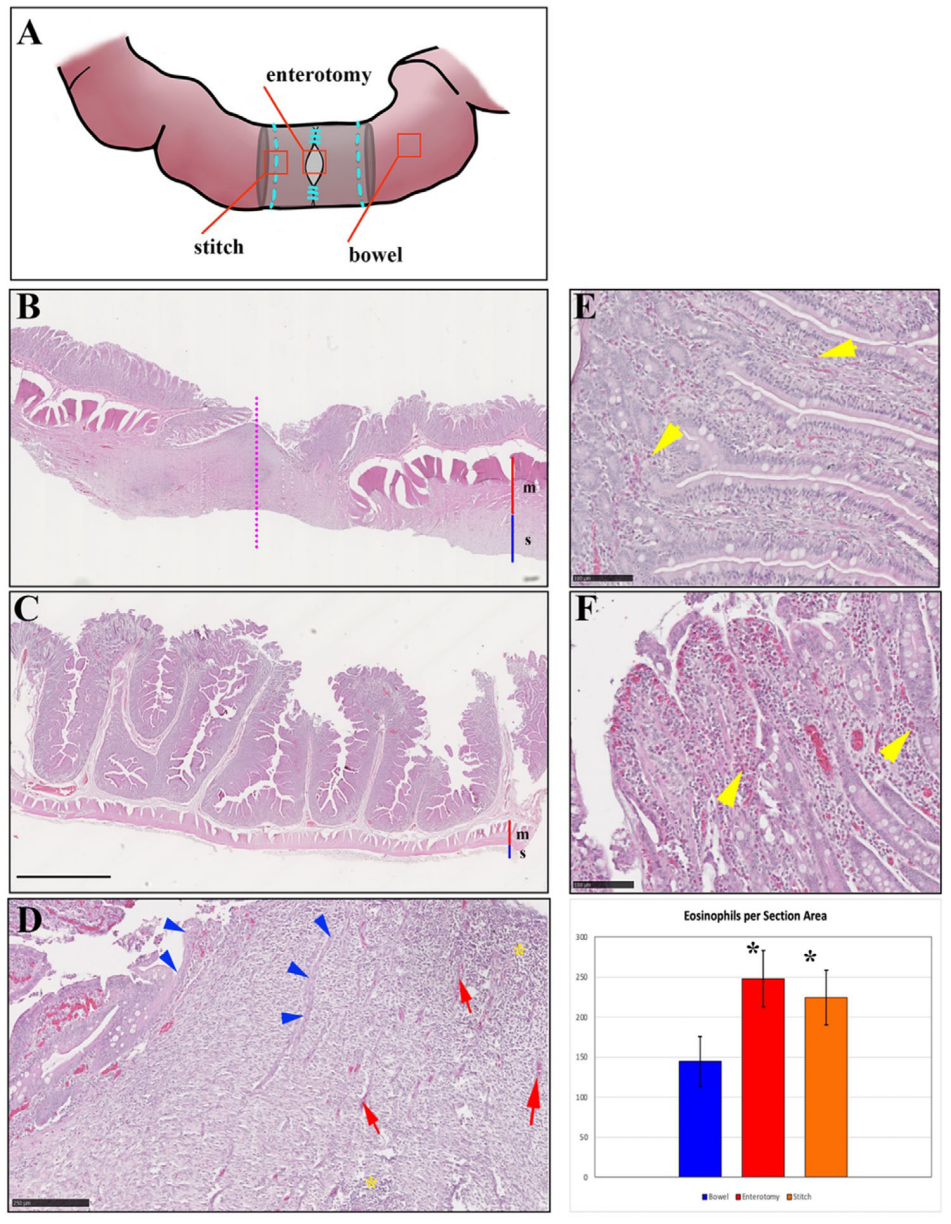
Histology of bowel segments. (A) Schematic diagram visualizing regions of the bowel taken for histological analysis, including the enterotomy, the area where the stent is anchored to the intestine (stitch), and an undisturbed region downstream from the stent (bowel). (B) Longitudinal section of intestine across the enterotomy (dashed line) showing thickened muscular and serosal layers compared to a region of the bowel downstream from the site of stent implantation, (C). Note also that the mucosal folds are flattened. Bar (B, C): 1 mm. (D) Region of the enterotomy, showing healing tissue reforming mucosal structures (blue arrows), blood vessels (red arrows), and areas of immune cell infiltration (asterisks). Bar: 250 mm. (E) Eosinophils can be observed in the mucosa of unperturbed sections of the bowel (yellow arrows). The presence of eosinophils (yellow arrows) is notably increased in the mucosa of segments implanted with stents (F), and in sections from the stitch region. Bar: 100 mm. The graph displays the number of eosinophils found by analysis of sections from the three regions analyzed. A significant increase in eosinophils (asterisk) is present in sections from the stitch and enterotomy regions compared to the undisturbed bowel, but there is no significant difference in eosinophil counts between enterotomy and stitch regions.

Histological examination of bowel samples from the region of the stent implantation shows healing of the enterotomy line, although reconnection of the muscularis and formation of intestinal mucosa over the enterotomy has not yet been reestablished (Figure 4). The region of the enterotomy has the appearance of granulation tissue that is attempting to reform mucosal structures. The muscularis mucosae and serosa [42, 43] are hypertrophied adjacent to the enterotomy, and the mucosal folds are flattened. Mild to moderate foci of immune cell infiltration can be observed in the serosa adjacent to the enterotomy and in the enterotomy region, indicating a mild inflammatory response. This infiltration is not observed in the mucosa, however a significant infiltration of eosinophils [44, 45] that can be found in mucosal tissue adjacent to the enterotomy. By counting eosinophils present in 20 random fields from each region, we found a statistically significant difference in eosinophil numbers according to tissue region examined (F(2) = 18.615, *p* < 0.00001). A Tukey post‐hoc test revealed significant differences between bowel and enterotomy regions, with an average difference of 103 more eosinophils per subsection in the enterotomy (*p* < 0.0000015), and between bowel and stitch regions, with an average difference of 80 more eosinophils per subsection in the stitch region (*p* < 0.00005). The difference in eosinophil numbers between enterotomy and stitch regions was not statistically significant. Sections of bowel tissue from the region where the stent was sutured in place show similar histological features, although the density of infiltrating immune cells in the serosa is more pronounced, presumably due to the presence of suture material in and near these sections.

**FIGURE 5:**
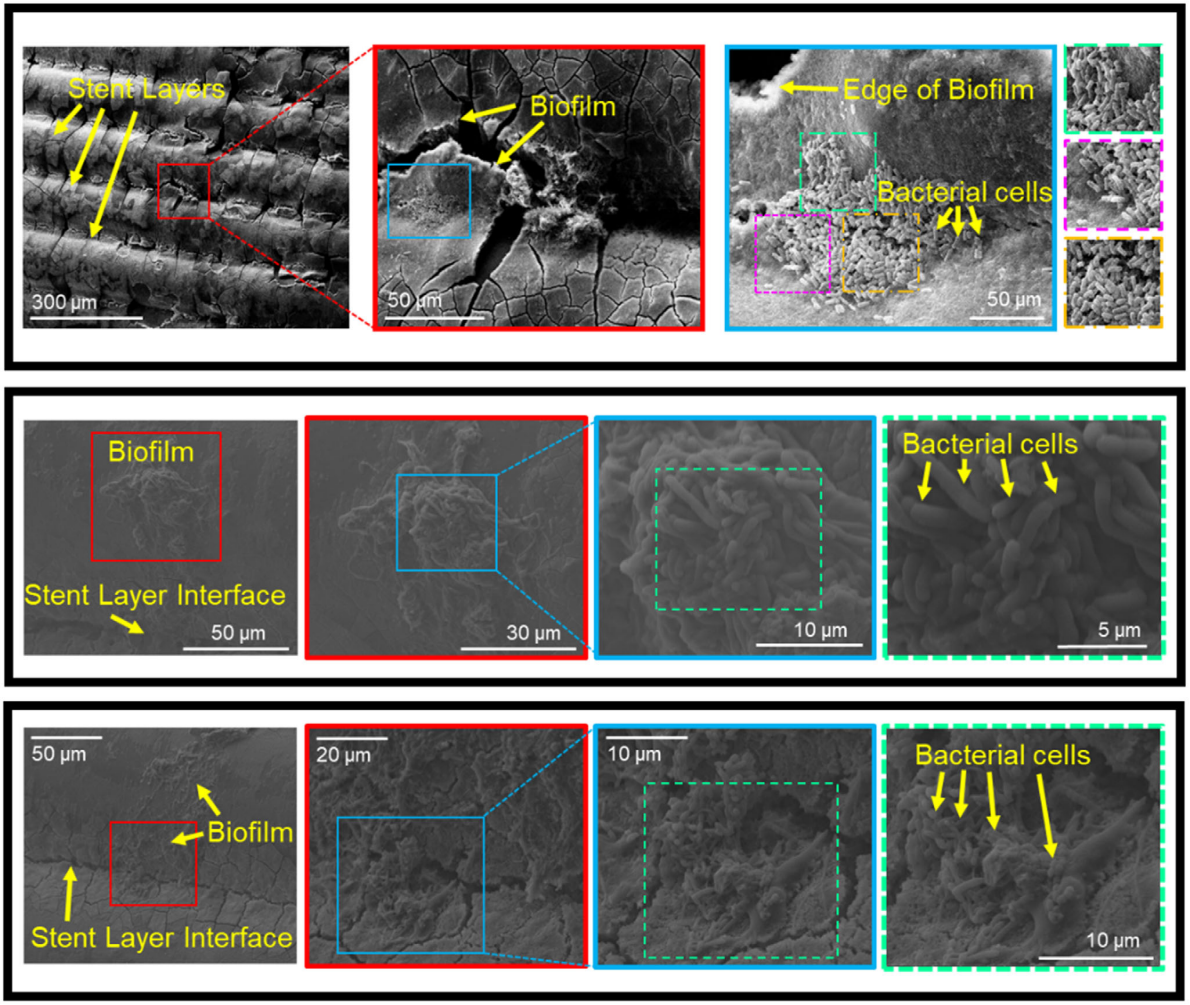
Scanning electron microscopy (SEM) micrographs of explanted stents. The stents did not exhibit signs of mechanical failure two weeks after implantation. Additionally, the stents were found to have been colonized by bacterial biofilms formed by the intestinal microbiota.

SEM images of explanted stents reveal that the stents remained intact while in the intestines and did not experience any apparent mechanical failures. Additionally, the presence of bacterial biofilms on the explanted stent surfaces (outer and inner) were observed (Figure 5). There were no indicators of stent bulk biodegradation observed in the SEM images.

## 3 Discussion

In this study, we demonstrate that 3‐D printed PLA stents prevent abdominal sepsis in a small bowel perforation model in swine. All four pigs survived until the planned euthanasia at 2 weeks, despite having a 1 cm enterotomy left at the site of the stent. One pig did have a fever on post‐operative days 1 and 2, however this resolved by post‐operative day 3, and it then returned to normal activity and interest in food. These findings are bolstered by the necropsy findings. There were no abscesses, signs, or bowel leakage or succus within the abdomen found on necropsy. There were significant filmy adhesions noted within the abdomen, particularly around the site of the stent, however they were consistent with post‐operative day 14 adhesions. In three of the four pigs, a loop of small bowel had adhered to the enterotomy site, while in the fourth pig the enterotomy site was adherent to the wall of the stomach. In all pigs, the stent appeared to prevent clinically significant leakage from the enterotomy and allowed for healing at that site. This is significant, as a 1 cm enterotomy would lead to intra‐abdominal sepsis and death if untreated. In humans, other treatments for an unclosed enterotomy include stoma creation or bowel resection, neither of which were applicable here. No control group was possible for this study as it was deemed inhumane to create and then leave a 1 cm enterotomy untreated. Overall, these stents prevented intra‐abdominal sepsis that would have otherwise occurred in all four pigs.

The clinical courses of all four pigs were generally unremarkable, aside from an initial fever which resolved in one of the pigs, and dark stools in all pigs at around post‐operative day 10. The etiology of the dark stool is unclear, and it resolved spontaneously after a few days. It is highly likely that bleeding at the enterotomy site caused the dark stool, but further investigation would be necessary to confirm this hypothesis.

Stent migration may have happened in pig 4. This enterotomy was adherent to the stomach and it was difficult to remove the small bowel and stomach during the necropsy due to the adhesions surrounding the enterotomy site. The stent appeared to be in appropriate position on the CT performed at one week, however it may have migrated in the second week before the necropsy or been inadvertently moved by manipulation of the bowel during the necropsy. Regardless, there was no sign of succus, abscess or intra‐abdominal infection on necropsy. This suggests that the anastomosis needs to be protected for less than two weeks for healing to occur.

The gut microbiome plays an important role in maintaining health, and disturbances in the gut microbiome have been linked to a variety of systemic and gut‐related diseases. The observation of bacterial colonization on the stent surfaces indicates that bacterial biofilms formed on the inner and outer surfaces of the stents after placement in the intestines. Additionally, since the pigs did not exhibit signs of infection, it can be inferred that the bacterial biofilms formed on the stent surfaces were formed from intestinal bacteria, and not by the introduction of bacterial cells via the stents. This observation is of interest in order to understand how the placement of stents can impact gut microbiota homeostasis [46]. We previously reported that 3D‐printed stents can provide a surface upon which intestinal cells can grow [21], which can assist in the intestinal healing process. The formation of biofilms on the 3D‐printed stents indicates that the stent surfaces are also amenable to the growth of native bacteria, thus minimizing negative effects that could be caused by disturbing the intestinal microbiome, and enabling the stents to integrate with the surrounding tissue microenvironment [47]. To the best of our knowledge this is the first example of PLA functioning as a scaffold supporting bacteria growth. A recent study investigating the effect of PLA microplastics (MP) on gut microbiota showed that PLA MP does not clearly alter microbial community homeostasis but showed increased levels of bifidobacteria according to 16S rRNA gene‐based metagenomic analysis [48]. Normally intestinal epithelial cells are covered with a mucus layer, which allows for bacterial adhesion and colonization [49]. Bifidobacterium are capable of producing molecules that promote adhesion and colonization of the intestinal mucosa in the absence of an epithelial or mucus layer [50]. Field emission scanning electron microscopy showed presence microbial biofilms on the polymer surface as well as changes to the surface morphology after treating PLA pellets (about 3.4 mm in size) in vitro with human colonic microbiota. Changes in the ester vibrations of PLA by Raman spectroscopy were detected as well. Based on these results the authors suggested that the human microbial community might be responsible for biotransformation of PLA. This is an interesting suggestion that might lead to faster degradation of the PLA stent compared to hydrolytic processes alone.

Histology shows increased presence of eosinophils at the enterotomy site. These innate immune cells participate in host immunity and help to maintain intestinal epithelial homeostasis [45]. The increased presence of these cells show that they have been recruited to the enterotomy site to fight inflammation. Histology data demonstrates healing at all enterotomy sites, with all pigs surviving until the planned necropsy at 2 weeks without showing sepsis or severe illness. Although there were adhesions from either small bowel or stomach to all enterotomy sites, there was no sign of fistula formation or abscess formation. This suggests that small bowel stents can be used to protect tenuous anastomoses and allow them to heal.

With the use of 3D‐printed stents, one additional consideration is the potential effects of 3D printing artifacts on the stent performance. For example, fused deposition modeling results in the presence of visible ridges for each layer in the stents. These ridges potentially present an increased surface area for the stents, which may affect degradation, the adhesion of mammalian and bacterial cells, and mechanical properties. Future work should be conducted to evaluate the effects of parameters such as print layer height and stent surface treatment (e.g. sanding) on cell adhesion, stent degradation, and mechanical performance. This is important because an ideal stent should be strong but flexible to avoid breakage resulting in the subsequent release of intestinal contents leading to sepsis, should integrate well with the intestinal tissue to provide a surface for intestinal healing, and should eventually degrade to prevent the need for later removal.

## 4 Conclusion

Our research team has created a stent made of PLA that can be surgically placed in the small bowel. PLA is a polymer that is already approved for medical use by the FDA. PLA is known to degrade over time by hydrolytic processes but bulk degradation of the stents was not observed on the timescale explored in this work, which aligns with previous reports that indicate a longer degradation time for PLA [51]. This paper reports a proof‐of‐concept animal study that demonstrates efficacy and short‐term safety in a swine small bowel perforation model. While longer‐term safety and efficacy studies are necessary, the present results indicate that 3D‐printed stents could provide a novel and impactful solution for a relatively common, difficult to manage, and devastating clinical problem. Although no degradation was observed within the timeframe of our study, the observation of bacteria on the surfaces of the explanted stents suggests that the stents were populated with intestinal bacteria. This may accelerate stent degradation, and further studies will be needed to assess whether long‐term degradation of these stents occurs and to confirm that it does not result in toxicity. These studies would also determine long‐term efficacy of the stents, and whether there are any changes in the gut microbiome because of stent placement.

## 5 Experimental Section

### 5.1 Materials

Commercially available PLA filament (Metallic silver color, 103 MPa flexural strength, 5.1 kJ/m^2^ impact strength, 83 Shore D hardness, a melt flow rate of 6.1 g/10 min at 210°C, and melting temperature of 145°C) was purchased from Ultimaker. This brand and color of filament have been used in the past for in vivo and in vitro studies, which have demonstrated its biocompatibility [52, 53, 54, 55]. The safety data sheet does not list any additives for this color of filament. Stents were 3D‐printed on an Ultimaker 2+ 3D printer.

### 5.2 Computed Tomographic Imaging

High‐resolution CT data were acquired on a Philips Precedence (Brilliance) 16 channel CT imaging system with X‐ray tube settings of 90 kVp and 800 mA. With a 100 mm field of view and 0.80 mm slice thickness, the length of the scan was 234 mm. The high‐resolution scans were acquired with a collimation of 16 × 0.75, pitch of 0.313, and a rotation time of 1.5 s.

### 5.3 3D‐Printing

**FIGURE 6:**
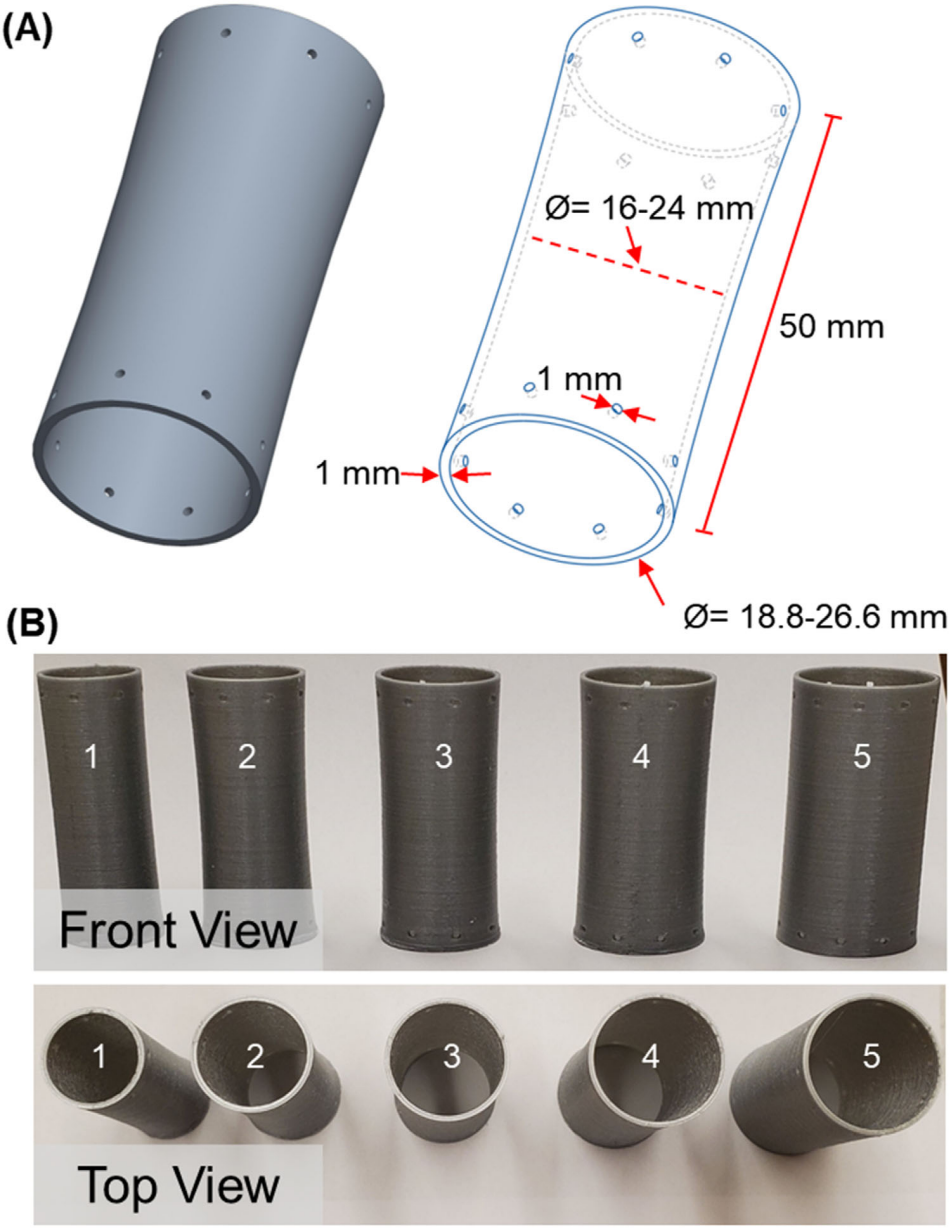
Gastrointestinal stents. (A) Computer model of stents. A curved stent design was employed, with a stent length of 50 mm, thickness of 1 mm, maximum outer diameters ranging from 18.8 to 26.6 mm, and minimum inner diameters ranging from 16 to 24 mm. Multiple 1‐mm diameter suture holes located near the ends of each stent allow for ease of suturing. (B) 3D‐printed stents in 5 sizes determined based on CT imaging of pigs.

Computer models of the stents were created in Creo Parametric software (Figure 6A). Stents were fabricated with a curved design as previously reported [21]. A thickness of 1 mm and total length of 5 cm was used for each stent. A range of relevant stent diameters was determined from CT images of the pigs prior to stent placement. Stents (size 1 to 5) were printed with maximum outer diameters of 18.8, 21.8, 23.22, 25.4, and 26.6 mm, with minimum inner diameters of 16, 18, 20, 22, and 24 mm respectively (Table 2). Four stents were printed for each of the stent sizes 1–4, and 3 stents of size 5 were printed. To allow for suturing, 4 to 10 equidistant 1‐mm diameter holes were incorporated 3.5 mm from the ends of each stent. The stents selected for placement in vivo each had 8 suture holes.

**TABLE 2:** Dimensions of 3D‐printed stents.

Stent Size	Minimum Inner Diameter [41]	Maximum Outer Diameter [41]	# of suture holes
1	16	18.8	4
2	18	21.8	8
3	20	23.22	8
4	22	25.4	8
5	24	26.6	10

The models were sliced into gCode files for 3D printing using Cura. The slice parameters included a layer height of 0.15 mm and nozzle size of 0.6 mm. Stents were printed with a nozzle temperature of 200°C to 210°C and printer bed temperature of 60°C. The sharp edges of stents were rounded using sandpaper. Photographs of 3D‐printed stents of each size are provided in Figure 6B.

### 5.4 Stent Placement

For these experiments, Yorkshire crossbred pigs weighing approximately 120 kg were used Under protocols approved by the University of Illinois Institutional Animal Care and Use Committee (IACUC # 19267). Prior to implantation of the stents the pigs (n = 4) were sedated with a ketamine, tiletamine and zolazepam, xylazine cocktail [56, 57] and imaged With CT to determine appropriate sizes for stent creation. The day prior to stent implantation the pigs were fasted. On the day of stent implantation, general anesthesia was administered to the pigs with a preoperative antibiotic. A mini midline laparotomy, an 8–10 cm incision was made in the abdomen to gain access to the peritoneal cavity, was used to access the small bowel. A small enterotomy, a surgical incision (2–4 cm in the small bowel, was made and the stent placed Within the bowel lumen. Stents were sterilized in iodine solution immediately prior to placement. Metal vascular clips were placed on the stent as well as on the sutures holding the stent in place to allow for post‐operative radiographic identification of the stent. 3‐0 PDS was used to suture the stents in place in a circumferential interrupted fashion Around both ends of the stents. The enterotomy was then repaired with interrupted sutures of 2‐0 silk, however a 1 cm enterotomy was left on the antimesenteric side of the small bowel. The small bowel was returned to the abdomen, the mini laparotomy was closed, and the animals were then allowed to recover from general anesthesia. The pigs were monitored closely for the next two weeks With a CT scan after one week to evaluate the stent and were kept on a liquid diet throughout this period. At two weeks, the animals were euthanized and necropsy performed. The abdomen and bowel were clinically evaluated, and the bowel with the stent in place was removed. Histology was performed on the bowel at the site of the enterotomy. The stent was extracted and scanning electron microscopy was performed to assess the ultrastructure of the stent

### 5.5 Histology of Bowel Tissue Samples

After removal of the stent, bowel segments were fixed in 10% neutral buffered formalin for 48 h, then processed for paraffin embedding using standard methods. Samples were trimmed and embedded in paraffin blocks, and sections were cut using a Leica rotary microtome set at a section thickness of approximately 6 µm. Samples were oriented to produce longitudinal sections of the bowel traversing the enterotomy. Sections were stained using hematoxylin and eosin and visualized using a Hamamatsu Nanozoomer digital pathology slide scanner.

### 5.6 Scanning Electron Microscopy (SEM) of Explanted Stents

Immediately after explantation, stents were lightly rinsed with saline, fixed in paraformaldehyde solution (4% by volume in PBS) for 80 min at room temperature, and then immersed in a sequence of ethanol/water solutions with increasing ethanol concentrations (50% v/v, 75% v/v, 95% v/v, 100%) for 10 min each. Following the 100% ethanol solution, stents were removed and allowed to dry under ambient conditions. After complete drying, stents were coated with a thin layer of platinum‐palladium and imaged on a FEI Quanta FEG 450 ESEM microscope.

### 5.7 Statistical Analysis

Eosinophil counts were quantified from 20 randomly selected microscopic fields within each of three regions: the enterotomy site, the stitch region where the stent was anchored, and an undisturbed bowel segment. To assess overall differences among these regions, an analysis of variance (two‐tailed F‐test) with fixed effects, main effects and their interactions was performed with the significance threshold set at α = 0.05. Data are reported as mean ± standard error of the mean (SEM). When the F‐test indicated significant variance among groups, Tukey's post hoc test was applied to determine pairwise differences (α = 0.05) between regions.

## Funding

Carle Foundation Hospital, Urbana, Illinois, USA

## Conflicts of Interest

The authors declare no conflicts of interest.

## Data Availability

The data that support the findings of this study are available from the corresponding author upon reasonable request.
